# Using normalization process theory to inform practice: evaluation of a virtual autism training for clinicians

**DOI:** 10.3389/frhs.2023.1242908

**Published:** 2023-12-19

**Authors:** Belinda O’Hagan, Marilyn Augustyn, Rachel Amgott, Julie White, Ilana Hardesty, Candice Bangham, Amy Ursitti, Sarah Foster, Alana Chandler, Jacey Greece

**Affiliations:** ^1^ Division of Developmental and Behavioral Pediatrics, Department of Pediatrics, Boston Medical Center, Boston, MA, United States; ^2^ Center for Continuing Education, Boston University Chobanian & Avedisian School of Medicine, Boston, MA, United States; ^3^ Department of Community Health Sciences, Boston University School of Public Health, Boston, MA, United States

**Keywords:** implementation research, normalization process theory, qualitative evaluation, autism, extension for community healthcare outcomes

## Abstract

**Background:**

There is growing demand for developmental and behavioral pediatric services including autism evaluation and care management. Clinician trainings have been found to result in an increase of knowledge and attitudes. This study utilizes Normalization Process theory (NPT) to evaluate a clinician training program and its effects on practice.

**Methods:**

The year-long virtual training program about autism screening and care management included didactic portions and case presentations. Focus groups and interviews were conducted with primary care clinicians (*n* = 10) from community health centers (*n* = 6) across an urban area five months post-training. Transcripts were deductively coded using NPT to uncover barriers to implementation of autism screening and care, benefits of the training program, and areas for future training.

**Results:**

Participants were motivated by the benefits of expanding and improving support for autistic patients but noted this effort requires effective collaboration within a complex network of care providers including clinicians, insurance agencies, and therapy providers. Although there were support that participants could provide to families there were still barriers including availability of behavior therapy and insufficient staffing. Overall, participants positively viewed the training and reported implementing new strategies into practice.

**Conclusion:**

Despite the small sample size, application of NPT allowed for assessment of both training delivery and implementation of strategies, and identification of recommendations for future training and practice sustainability. Follow-up focus groups explored participants' practice five months post-program. Variations in participants' baseline experience and context at follow-up to enable application of skills should be considered when using NPT to evaluate clinician trainings.

## 1. Introduction

For the past decade, autism diagnosis and demand for developmental-behavioral pediatric care has been steadily rising with the most recent autism prevalence reported at 1 in 36 children (1). Increased demand of developmental services combined with disinvestment (2) and disruptions from the COVID-19 pandemic (3) has led to service delays. This is problematic given that benefits of early autism diagnosis and evidence-based care are well established (4, 5). Although screening is recommended to occur between 18 and 24 months of age (6), most children receive diagnosis after their third birthday, which is the cutoff for state-funded early intervention programs (7). Access to autism developmental care is especially strained in low-income and minoritized communities partly due to shortage of pediatric specialists (8).

A proposed strategy to address gaps in services is training more clinicians to conduct developmental screening and evaluations (9) instead of waiting for referral to specialists. The Extension for Community Healthcare Outcomes (ECHO®) has shown promise in equipping primary care clinicians (PCC) with skills and knowledge on a variety of clinical areas (10, 11) including autism. The ECHO model consists of a Hub institution comprised of topic experts responsible for delivering trainings and Spoke institutions comprised of PCCs (10) less expert in the topic. Researchers found that autism-focused ECHO programs resulted in increased participant knowledge and confidence (12–16). For example, Mazurek et al. (2019) found that a 12-month ECHO program increased clinicians' ability and self-efficacy to screen for autistic patients in Missouri, United States (13). There has been little evidence, however, that autism-focused ECHO programs result in practice change (17). Most studies on ECHO programs focusing on autism used a pre/post design but did not qualitatively assess practice change at follow-up (13–15, 18–20). Evaluating both pre/post knowledge/skills changes and application of practice within the service delivery setting are necessary to understand training impact.

The current study uses Normalization Process Theory (NPT) (21) as a framework to guide the Boosting Capacity to Screen and Care for Underserved Autistic Children ECHO Program (BCAEP), a training designed to enhance autism screening and care in primary care settings. The BCAEP training was conducted virtually during the COVID-19 pandemic and supported PCCs during major care disruptions. NPT posits that there are four components of practice change, namely coherence (i.e., what the new practice is), cognitive participation (i.e., one's and others' roles in the new practice), collective action (i.e., steps needed to accomplish the new practice), and reflexive monitoring (i.e., evaluation of the new practice) (21). NPT emphasizes individual and collective behaviors, in addition to attitudes and beliefs, which aids practice-focused queries (22). Past studies have utilized NPT to assess new practices in healthcare settings (22, 23). This study advances previous research by applying NPT to evaluate delivery and effectiveness of PCC trainings in urban safety-net settings as well as the extent to which learned skills and knowledge are applied in practice months after the training.

## 2. Methods

### 2.1. Program description

The BCAEP training was conducted virtually between November 2020 and October 2021 over 12 60-min sessions. Participants (*n* = 47) represented PCCs in seven health centers in the Greater Boston Area caring for safety-net populations. Trainings were facilitated by a senior developmental behavioral pediatrician with over 30 years of experience and an advanced practice clinician with 19 years of experience; both were affiliated with a safety-net academic medical institution. Each session consisted of didactic lectures and deidentified patient case discussions as per the ECHO model (10). Topics of the didactic lectures were determined based on an initial participant survey and were adjusted according to participants' interests. Examples of lecture topics were administration of different autism screening tools, engaging with patients in a culturally sensitive manner, and communicating an initial autism diagnosis to families.

### 2.2. Study design

This study was part of a larger mixed methods evaluation of the BCAEP training and represents the qualitative component of the evaluation. Focus groups and interviews with BCAEP PCCs (*n* = 10) were conducted to contextualize quantitative survey responses and provide actionable practice recommendations. Participants were asked to complete a brief pre-focus group assessment that gathered information about their practice (Table 1); nine responded to the survey and ten participated in focus groups. There were 30 pre-test (before first session), 19 mid-point (after sixth session), and 17 post-test (after twelfth session) survey responses, with nine matches from pre-test to post-test. Survey findings indicated an increase in participants' reported knowledge and self-efficacy in administering autism screeners and managing care in post-test compared to pre-test. Each of these quantitative findings warranted further exploration qualitatively and at a time when participants had a chance to implement the training learnings in practice.

**Table 1:** Focus group and interview participants characteristics (*N* = 9)^a^.

Are you attending this focus group in-person or virtually?	
In-person	1 (11.11%)
Virtually	7 (77.78%)
Neither: I am participating in a personal interview	1 (11.11%)
What is your title/role?	
Physician	4 (44.44%)
Nurse Practitioner	4 (44.44%)
Administrative leadership	1 (11.11%)
Number of years in practice	
Less than 2 years	0 (0.0%)
3–5 years	3 (33.33%)
6–10 years	1 (11.11%)
11–20 years	3 (33.33%)
21 + years	2 (22.22%)
What is your medical specialty?	
Pediatrics	7 (77.78%)
Psychiatry	1 (11.11%)
Other (integrated behavior health)	1 (11.11%)
How many ECHO sessions did you attend?	
Mean (SD)	10.1 (1.96)
Min, Max	6, 12
Are you currently implementing strategies (e.g., first-level/second-level screeners, patient education, resources) from ECHO Autism in your practice as a clinician?	
Yes, all strategies	4 (44.44%)
Yes, some strategies but not all	4 (44.44%)
No, I'm not implementing any strategies	1 (11.11%)
Is your clinic currently implementing strategies (e.g., first-level/second-level screeners, patient education, resources) from ECHO Autism as part of clinic protocol?	
Yes, all strategies	2 (22.22%)
Yes, some strategies but not all	7 (77.78%)
No, my clinic is not implementing any strategies	0 (0.0%)
I don't know	0 (0.0%)
Were there other providers/staff members in your clinic who also participated in this ECHO Autism training program?	
Yes	9 (100%)
No	0 (0.0%)

^a^ One participant did not complete the pre-interview survey. Therefore, nine participants were included in this table and ten participants participated in the focus groups.

Focus groups and interviews were conducted virtually approximately 6-months post-training using Zoom or in-person depending on participants' availability. This study was reviewed and approved as exempt by the Boston University Institutional Review Board (IRB #H-40718).

### 2.3. Participants

Participants for the qualitative assessments were recruited through convenience sampling of PCCs who attended the training. Participants represented PCCs from six out of seven (85.7%) eligible health centers. Recruitment emails consisting of abbreviated consent forms were sent. Participants were asked to complete a brief pre-focus group assessment that gathered information about their practice (Table 1).

### 2.4. Data collection

Semi-structured focus groups and interviews were conducted in March 2022 using a guide (Appendix A) to gather information on individual level and clinic-level application of strategies taught in the BCAEP training as well as opinions about the training delivery. Questions were informed by survey findings, NPT constructs (21), and outcomes indicated on the logic model. Focus groups were conducted by two evaluation team members; a lead moderator and note-taker. One member was present at all focus group and interview sessions for consistency. Audio recordings were transcribed and verified by two different members for accuracy.

### 2.5. Analysis

Deductive coding (24) using NPT constructs (21) and content analysis (25) were conducted on six transcripts. The coding team consisted of three evaluation team members who were trained to conduct qualitative analysis using NVivo12 (26). Each transcript was coded by two members. All three members then met to discuss discrepancies by consensus with the third member serving as tiebreaker if consensus was not reached.

## 3. Results

From the nine pre-focus group assessments, participants included physicians (*n* = 4), nurse practitioners (*n* = 4), and administrative leadership (*n* = 1) with most participants having 3–5 years (*n* = 3) and 11–20 (*n* = 3) years of experience. Specialties included pediatrics (*n* = 7), psychiatry (*n* = 1), and integrated behavior health (*n* = 1). On average, participants attended 10 out of 12 training sessions. Themes were organized by NPT constructs and presented with frequencies and illustrative quotes (Table 2) in order to provide a framework for actionable recommendations.

**Table 2:** Qualitative findings from focus groups and interviews with primary care clinicians organized using normalization process theory.

Construct	Definition	Illustrative Quotes	Frequency
**Coherence** is the initial sense-making work that is involved in ultimately implementing new tools and methods in practice. This sense-making involves both individual and communal aspects, such as individuals defining internal motivation for learning about these new practices, as well as identifying their roles as individuals and within a community of providers as they begin to think about how these practices will realistically be applied.			
Differentiation	Participants identified how strategies and protocols discussed during the training sessions differed from their current practice. Examples include referral protocols, addressing behavior in different demographic groups (e.g., children of different ages), and implementing available assessment tools at different times.	**109**: **“**Listen, it is glaringly obvious that this is a child who has autism. And in the old model, we could put in a referral to [outside institution] developmental-behavioral peds, and know that eventually they get to a great clinician who's gonna do the assessment, get that diagnosis, and then get them the services that they need. But that bucket of kids might be better served if we can keep them sort of in-house with the [community health center]'s team and use these tools that, you know, [psychiatrist] was using…. There [are] probably some kids that the PCPs more and more could and should be doing those tests, and that's what [my clinic] is trying to, to do moving forward.” **102**: “Uh having like more concrete examples, umm maybe also seeing the different kinds of, the videos, for instance kids of different ages and genders and seeing some of those behaviors can like bring it back and see how, you know, I remember seeing a certain example and so when you see it again that's helpful too.” **109**: “like, don't see the kid and then finish it, and then wait to score your CARS like a week later. Like it is really important, do it right away when it's fresh, and that was something I, I directly tr—um, took, and have been trying to do a much better job about doing.”	4
Internalization	Participants shared the internal motivations that they had to engage in the BCAEP. Generally, participants found the BCAEP valuable as it reinforced some of the concepts that they had learned about in previous training. Many participants already had a particular interest in autism and developmental screening prior to completing the training, especially considering that they are seeing more patients with autism-related concerns, and because of influence from peers who had previously participated in other ECHO programs and had positive experiences.	**107**: “I mean I think I… completely learned some things that I had half learned… and I unlearned a couple of things that were that were wrong……. I had a little bit more appreciation of the evaluation, although I… learned that on top of experience with those evaluations from 20 years ago……. I learned how things had been refined…. I guess for me it was updating mostly’ … cause I had put some effort, some misplaced effort into it before in my career and… kind of cycled back to it a little bit more.” **110**: “And I think part of the reason I feel comfortable doing this training and then starting that primary care is that a lot of my team members have done the ECHO training and felt like it, like, feel even more confident in being the person to diagnose autism and talk about next steps and help support them with management afterwards.” **108**: “…I’ve been in practice for 25 years. And I never had this many autistic kids, and I’m not talking about the subtle ones, I’m talking the ones that are so obvious. I don't know what's going on but it's really, it's really taken up a great part of my practice, so this was terrific.”	42
Individual Specification	Participants noted their individual tasks and responsibilities related to autism healthcare and diagnosis. Participants’ roles varied such as medical director, nurse practitioners, and other clinicians. There appeared to be two categories of participants: (1) specialists who can use the information that they learned in BCAEP to strengthen the practices that they already had in place, and (2) primary care clinicians who do not necessarily specialize in autism but can use the information from BCAEP to better screen for and follow up with autistic patients, rather than always having them see a specialist. A strong theme of differences between healthcare and ABA providers was discussed in multiple focus groups, and how it might be helpful for healthcare clinicians to learn more about ABA so that they can implement elements of it in their medical practice.	**101**: “… I would have loved to… [learn more about] the ABA portion itself. I find it very difficult to address [ABA] with parents… because… I don't really know how ABA. I mean I have an idea. But really, because parents ask and say ‘Okay, what is ABA and how is it going to get better with ABA?’ And that's something I’d love to try to explain better…” **105**: “So I think that all of it was… more learning for me. Because we don't work with autism, so… it was a way of getting more than just reading, more than just book knowledge, and thinking about what [we are] looking for. So, for the team that's working with the [young children], or even someone who is a teenager: what are those… signs we might want to be noticing that we can then loop back and say, ‘Hey, we’ve been spending this amount of time, and this is what we noticed’. And so, for me, this is about how do I take that information from the trainings and put it into some practice?” **105**: “Our family partners are pretty amazing in the work that they're doing. And I would love to learn more about what Early Intervention is doing on… Are there these little tidbits that we can do in primary care? We’re not gonna be able to do what in-home… or true ABA or anything like that [does]. But are there tools and small things that we can support parents on doing to help their child in the interim?”	34
Communal Specification	Participants discussed coming to a shared understanding of objectives with their colleagues, patients, and families. Participants discussed how to collaborate with colleagues to improve clinical systems and be on the same page about a patient's diagnosis. Participants found it useful to hear about challenging cases that came up in other healthcare centers and how other clinicians addressed the situation in informing their own understanding of these screening tools. Further, participants expressed a desire to better understand the roles of others acting in the field (e.g., pediatricians, ABA providers) to work toward a common goal.	**105**: “Or if parents are expressing a concern being able to support or follow through on the conversation the pediatrician is having. So, we’re not diagnosing but we’re at least being able to provide bidirectional information. And I think that's what really important.” **109**: “I think that I’ve felt more confident through hearing other health centers talk about maybe those challenging cases…. How do you talk about what ASD [autism spectrum disorder] is…. When we look at it through a lens of a biomedical sort of Western medicine-informed diagnostic entity…. but I’m seeing a newly immigrated from Albania family that doesn't speak English… In their culture, there's an idea that… not talking until the age of five is actually pretty typical, and [the family is] not concerned about that at all…. We need to somehow meet each other in the middle… and do what we think is in the child's best interest while honoring that family's values…. I think the ECHO was also really useful to hear different clinics talk about those types of dilemmas, and this is how I would talk to the parent about that…” **105**: “Being able to think and understand how pediatricians approach a patient, as well as the concerns and challenges they face in thinking from that behavioral health lens…. What could we do? What could we hold with them so they're not holding it alone?” **110**: “… I think we’re more able to discuss what ABA therapy looks like. I think… we’re getting trained on level two screeners in two weeks, and gonna hopefully be using that to do some diagnosis… onsite…”	18
**Cognitive participation** involves problem identification, collaborations within a clinic to work towards solving the problem, and how clinicians can confidently sustain their practices. Participants identified the pieces that need to come together to lead to positive change in their individual and clinic-level practice. This thinking involves identifying the individuals who will bring about this change and the context in which they will be doing so. Further, participants reflected on whether they are confident that they have the tools and people necessary to exact change			
Initiation	Participants recognize long wait times and gaps between appointments as barriers in their practice. Participants reflected on the gap between their current practice and new strategies introduced in BCAEP. Many BCAEP participants were building from practices that they already had in place following the training. However, others were just getting started with thinking about how to change their practice to better serve autistic patients. ** **	**109**: “… I guess when you go through med school, there's still definitely that lingering altruism of “see one, do one, teach one”. And I trained in the CARS as a fellow, probably, you know, actually performed it with some small [number of patients], and then I was in the real-world practicing and using it…” **107**: “… Compared to the other health centers… that were involved in the ECHO, [we are] so much bigger… that we should be better able to… make a change like that. Whether we actually are good enough to actually make a change like that, is… a whole different issue you know what I mean? But… we can make decisions that smaller health centers can't make….” **110**: “Oh, how I [determine whether new protocols are working for my community health center]? I would foresee that the majority of time when we have a child who has some developmental or other concerns that bring autism…—the child would have an answer, or at least a next-step plan within two months. You wouldn't have a child that's eighteen months old and wait for a diagnosis or at least for a next-step screener for more than two months. [Moderator 2: Mhm. Okay.] Or older child, but I’m thinking of eighteen months to three-year range.”	7
Enrollment	Participants discussed how they collaborate and learn within their health center, such as having screener trainings and having separate groups to target unique aspects of autism diagnosis/treatment. BCAEP solidified the desire of many participants to enroll additional individuals who previously did not screen to implement autism screening measures, for example, as well as to participate in the BCAEP itself.	**109**: “Listen, it is glaringly obvious that this is a child who has autism. And in the old model, we could put in a referral to [outside institution] developmental-behavioral peds, and know that eventually they get to a great clinician who's gonna do the assessment, get that diagnosis, and then get them the services that they need. But that bucket of kids might be better served if we can keep them sort of in-house with the [community health center]'s team and use these tools that, you know, [psychiatrist] was using…. There [are] probably some kids that the PCPs more and more could and should be doing those tests, and that's what [my clinic] is trying to, to do moving forward.” **106**: “As I said, we're sort of working on, we had just started trying to become more autism friendly and… then the pandemic came. So, I think that sort of a combination of that, plus the ECHO plus [clinician]'s mini fellowship. [Clinician] and I are working on making almost sort of a mini developmental clinic day type thing so that we have much better, we have more wraparound services than we had before.” **110**: “I wouldn't say that we’ve implemented any direct change yet. But we’re doing a training on April 13th with a… developmental pediatrician who was at [academic institution] and is now at [hospital] training our primary care providers in the RITA-T screener. [Moderator 2: Mhm] With the plan that we’ll start a practice of, um, using the RITA-T to decide who is—has a high enough score that that could be consistent with a diagnosis for autism and actually writing the letter to diagnose within our primary care practice, and who's in this kind of middle of the road area where maybe a developmental pediatrician”	14
Legitimation	The BCAEP increased participants’ confidence in implementing screenings and follow-up procedures. It also served as a reassuring place for healthcare professionals to be able to share feelings and ideas about challenging situations.	**106**: “I did start doing CARS evaluations after the program started, partially because I just had more confidence in doing it… The CARS doesn't have an official training *per se*. I think that part of the reason I started doing them during the program was cause I just, it gave me a little bit more confidence, and partially because that's just kind of when things fell into place.” **110**: “I think our clinicians are increasingly confident in using the autism word and saying autism is something I’m worried about, or autism is something I’m thinking about, or autism is something I think we need to evaluate for, which I think helps, um, I think—”	10
Activation	Skills learned in BCAEP fit into the bigger picture as they allowed individual clinicians to work together more efficiently and get crucial steps, such as an autism diagnoses, done more quickly. Looking ahead, participants feel as though BCAEP would prepare them to seek available resources to offer their patients and be more straightforward with patients about autism diagnoses.	**109**: “… I’ve been at [community health center] for three years. In the early months, it could feel like,… I might see a kid over three sessions get the assessment and the diagnosis done. Involve the referrals team, and it, it might be like four, five months until we feel like that kid finally gets connected with a, you know, ABA team. And that's the first way to answer your question is that I think we’ve gotten more efficient, and the diagnosis is done quicker…. I have some kids that the diagnosis letter is written, you know, within a week. I saw them, and the diagnosis is clear. We make the diagnosis, and then the referral to those services… through the community health worker team is happening faster, so that's one improvement is just the speed of all that.” **102**: “… Better able to talk about like what's available in the moment… For instance, Early Intervention for the younger kids or school and getting them set up at schools, and then again like the ABA for afterwards.”	9
**Collective action** refers to the action steps that participants need to take to implement new practices they learned from the ECHO training program. This involves interacting with the systems already in place (e.g., referral process), barriers (e.g., COVID), allocating time and resources, and building systems of accountability (e.g., defining staff roles and responsibilities) to ensure that these changes are implemented effectively.			
Interactional Workability	Autism diagnosis and follow-up care involves many players with whom BCAEP participants interact, from schools to insurance agencies to ABA providers and more. The efficiency of these interactions is a major factor in determining the ease with which the practices discussed in BCAEP can be effectively implemented. Participants noted about barriers in getting patients diagnoses after referrals and secondary screens due to long wait times, challenges with medical record systems (e.g., EPIC), billing delays, diagnoses being rejected, little collaboration across departments, and limited capacity of clinicians.	**107**: “… We still are stuck with this bewildering random delay as to whether basically billing agencies will accept those diagnosis. So, our patients all have the same MassHealth insurance. They all have C3… and so we'll refer someone to ABA through an agency that has a short waiting list. And they'll accept our diagnosis because they think that they'll get paid eventually through MassHealth. Then, they'll get a big waiting list, and we'll go to a different agency, and the different agency won't accept our diagnosis because they think they won't get paid through MassHealth. Now that's only because understanding what MassHealth will do and won't do is… a bewildering sort of mess. But we have the same person doing the same test with the same letter template… They're literally all… quite the same… and we get different responses… and that's because agencies don't know what's going to happen, right? And no agency is going to do… six weeks’ worth of work and then… feel like they're not going to get paid for it… Although… it would surprise me if any of the agencies could actually trace back any of their individual units of work to… real specific dollars that come in… for services because we certainly can't at the health center.## So… we have… no idea whether… we're being reimbursed for the service codes and… diagnosis… for the visits [and] for the screening that… we're doing.” **107**: “…Because beyond not being able to match people with ABA therapy… the real troubles that we've had over the last couple of years is that our local Early Intervention program has been almost missing in action. I mean… [state] school department certainly has been missing in action, right? [Moderator 1: yeah] So… we have to coordinate with those two agencies and…. I mean I… know why our local Early Intervention program was missing in action ‘cause it was all the same things that we were going through also…” **105**: “The [electronic health record (EHR)] is not talking to one another. [106: Your EHR doesn't talk to people? You don't have that?] 105: Haha, we have EPIC, but care everywhere has its limitations, right? So, if you refer to developmental peds, I'm not sure how quickly or what you're actually being able to see. And we have EPIC OCHIN. We're not fortunate to have EPIC EPIC, so. [106: You know, it's better than when the EMRs didn't talk to each other.] 105: True. True. (Laughing)” **106**: “Unfortunately, even when I was doing secondary screens and I was sending messages to [Nurse Practitioner] saying, you know, I just saw [patient] and her STATS screen, her secondary screen was like off the scale, it wasn't necessarily getting those kits in a whole lot faster because the system is the system, unfortunately.”	19
Relational Integration	Creating more structure and accountability within individual healthcare centers has allowed BCAEP participants to implement screenings and follow-ups more successfully and to quickly get autistic patients the services that they need. In contrast, lack of structure and accountability led to difficulties in screening and services enrollment. Participants found it helpful when additional colleagues were trained in CARS as it speeds up the diagnosis/treatment process.	**102**: “I would love somebody, this requires man power… But right now when you put in an ABA [referral],… let's say I make a diagnosis and write a letter support for ABA and I send a parent out and there's… nobody to support them. I know we have the autism team but they are now limited in how.., this is maybe internal to our clinic or our institution but… I would love someone to walk [families] through this [ABA] process because… you see them time and time after that and you’re like ‘Where are you in the process?’ ‘Oh,… we haven't really started or… we got stuck at paperwork which is… the very beginning [step]’…. But that may require funding for somebody in.. or, I don't know if there's an online format for someone.. some website that teaches you how to get that stuff that you need, you know even from EI, if not for the.. our patient navigators that are in clinic that are wonderful, and I message them cause… you refer a patient to EI and then like nothing happens.” **105**: “And then I would second what you said about the ABA and I’m trying to think about would it be beneficial for… some of our staff to be trained to some degree in it so that we can do some little tidbits of support.” **106**: “We’re continuing to have, unfortunately, a lot of trouble getting kids hooked in with ABA, and part of it is that MassHealth now is claiming that I am not qualified to do autism evaluations. [Moderator 1: Why is that?] I think that there may have been a misinterpretation at some point about part of their blurb which says “expertise” in developmental, does not say “specialize”, so we’re working on [Moderator 1: Huh.] arguing through that one.”	12
Skillset Workability	Overall, BCAEP participants expressed the hope to expand training for autism screening (CARS and RITA-T) beyond specialists and for primary care clinicians as well. When more providers can perform these screening procedures, the process is more time-efficient, and autistic patients can get the help that they need more quickly.	**109**: “… If it was all in the hands of one person to be responsible for all of those pieces, I think it can become a little unwieldy and time intensive. And then you’re back to the timeline of… we eventually got the kid where they needed to be, but that diagnostic journey took eight months, and that's eight months of their development… So that's the main thing is I think that [integrated behavior health program], multidisciplinary approach at [community health center] is gonna help us continue to improve our autism assessment process.” **109**: “There's talk at the clinic of expanding the training for other providers including the pediatricians themselves to become more skilled with the RITA-T and the CARS [Moderator1: Yeah]. Training them in how to do the diagnostic… letter… that insurance will accept… in order to get ABA, the IEP, all those services going… And that sort of to get better as a clinical team… [in] involving behavioral health staff, involving primary care provider, to… maybe catch those kids that fall under the bucket.”	28
Contextual Integration	Participants discussed barriers in implementing autism screening and follow-up measures in practice, such as short visit times, increasing gaps between screening checkups in pediatric populations, and inefficient recording systems. BCAEP sparked ideas in some participants for addressing these issues. When implementing of screening and referral protocols, clinicians expressed frustration in scheduling challenges, time-consuming and expensive nature of services (e.g., ABA, developmental evaluations). Difficulties in implementing autism screenings and follow-ups were even further exacerbated by the COVID-19 pandemic when in-person visits were limited.	**107**: “…One aspect of diagnosis is that the visit… schedule goes from every two months to… every year… very quickly….. Then what happens is that kid is too old for Early Intervention, [but] too… young for school…. And the specialists already have a one year waiting list for kids that are 19 months old…. So now, we want to build something in our practice to catch that… but we don't have enough kids in our practice to build that system—to run 1,000 kids through in that age group, you know what I mean? So… the math doesn't work for practices I guess.” **102**: “… We’re in kind of like a paper world. So, you get the paper screener… and then it's put into the computer, or you can yourself put it in or have your medical assistant [or] whoever. Sometimes there are lags or drops in [that] process. I think the ultimate goal and what most clinics should be really doing in this day and age is… everything should… just [be inputted] directly into the computer…. This is probably off topic, but a lot of practices are like having parents screen things prior or in the waiting room in a virtual kind of format. So, we weren't there yet so we were kind of talking about that process.” **103**: “I mean, I do think that… certainly a barrier is… time, right? I mean we… were scheduled with—I mean, for us in, at [community health center], the nurse practitioners get a little bit longer [visit time]. But even still people run late, it takes time to room, then you got your next patient waiting. And so, it's really hard. And… to do a full autism evaluation and ADOS or… whatever it is they're doing now, it's like an hour, right? And so, it's obviously really hard to… get a full picture of a kid's development in… a 15-minute timeframe. And so, I do feel like the training has helped in terms of making my time with a family more efficient and trying to figure out: Am I concerned? Am I not concerned? And… what is the concern?… Is it really just… a language delay or is there some additional piece, like the social communication, things like that?” **104**: “There's only three pediatricians and me… and we are teeny tiny. We have two exam rooms each, and we are all four of us in a tiny office. So, we don't have tool kits. I guess Dr. ### has her own, she's our newest… provider, but feel like we work in silos with regard to… autism, how we approach autistic kids because we have kind of systems in place…”	32
**Reflexive working** occurs when participants evaluate the training that they received as well as the implementation of strategies that they learned during the BCAEP.			
Systemization	Systemization was defined as how participants assessed utility of strategies learned during the training.	**102**: “…We kind of have some difficulties in getting screeners like input into EPIC [electronic medical record system] sometimes… so we try to work on that a bit more… After the ECHO [training]… just realizing… importance [of documentation] and trying to make maybe some flow changes or systematic changes to help us to really not miss these key… screeners.” **107**: “So we do have… a provider who has.. four to six hours a week to just do… the screening the screening diagnostic, the secondary screening diagnostic tests, wait, the secondary screening tests… Because we wanted to try to move kids faster through the early intervention… and school bottleneck… And that's working reasonably well… which is a big… improvement from… before… As a result of that I believe that we've been able to get kids seen at specialty programs or by neurologists more quickly than if we had not been doing that.”	42
Communal appraisal	Communal appraisal occurs when participants evaluate how a strategy learned from the training can be beneficial for their colleagues and families they served. It also occurs when participants reflect on the process of coming to a shared understanding of best practices with other clinicians in the training.	**102**: “…I mean, I think we got to know each other… you could tell… some personalities throughout the end for sure. And I love the fact that I could see into the thoughts and experiences of others in my very close vicinity that I never would have met, absolutely never would met otherwise especially post-pandemic… And to hear maybe some of what other practices are struggling with or doing well with so that whole like community feel I thought was great.” **103**: “…It's always nice to hear about how things run differently in different clinics, because you start to realize well, maybe we could be more efficient in this way. You know, we could… adopt some of the… processes that are used in other clinics. And so… I feel like it's nice, … not only for getting to know folks in the community, but also… sharing ideas about what's worked, what hasn't worked… in their clinics and ways to improve ours.”	31
Individual appraisal	Individual appraisal occurs when participants reflect on how the BCAEP benefit them as individual clinicians. Participants discussed recommendations to improve BCAEP training delivery.	**109**: “I valued… the case presentations. This is gonna be such a psychiatrist answer… we think about things like the process vs. the content. And the content was obviously very informative and useful and filed away in some way. But it's more… that one level up. The process of [going] through a case presentation from another pediatrician at another health center about a tough diagnostic disclosure with a family with language and cultural and whatever barriers. Like that experience… and that one level up learning… I found the most valuable.” **102**: “… I loved the videos, whenever those were brought in, I thought they were just such a great teaching tool like when [trainer] would show examples for instance of children that had autism or had of different ages. That was great because we obviously can't practice on Zoom or in these ECHOs but maybe a couple videos of observing different secondary screeners would be great, or screeners in general. I think just the video may be just a great tool.” **102**: “…I would love like somewhat, so um maybe screeners available either in a virtual format or paper format kind of stocked in every room.”	100
Reconfiguration	Reconfiguration occurs when participants reflect on current or future/planned changes to their practice because of the BCAEP.	**108**: “Just to agree with 101. After the… training, [I] made a point of using the word [autism]. And… I don't know if I was shy about it before or not, but what I learned was that most parents were thinking it anyway, and if you didn't say it, then they continued to worry inside that they might be crazy or something, because… almost none of them rebelled against it. You know, they almost all, like, appreciated hearing that.” **110**: “…So I guess the only thing I’ll say is that it made us even less tolerant of the wait times and moving more things and like doing more work to kind of move heaven and earth to not let the wait times be a barrier for our patients… That's probably the way that the ECHO impacted things.”	30

The following sections consisted of quotes coded with the corresponding constructs. NPT consisted of four constructs, each with four sub-constructs (21) that start with an understanding of the new practice (i.e., coherence), operationalizing change (i.e., cognitive participation), implementing the change (i.e., collective action), and ending with its evaluation (i.e., reflexive working) (22). While all constructs contributed to the findings and subsequent recommendations, reflexive working and collective action, given the role of clinicians to implement the concepts and assess the utility in practice, were most often mentioned.

### 3.1. Coherence

Coherence occurs when an individual attempts to make sense of the new practice (21). This construct manifests through an individual's perception on a practice as well as their motivation and role for the new practice implementation.

#### 3.1.1. Differentiation

In the current study, differentiation occurred when participants discussed how strategies taught in the training differed from their current practice (21). For example, one participant shared that “*in the old model, we could put in a referral to [external institution] developmental-behavioral peds”* but there are “*kids [who] might be better served if we can keep them sort of in-house.”* Another participant mentioned how behavioral observations may differ depending on the child's age and that “*having more concrete examples… maybe also seeing… the videos… [of] kids of different ages and genders*” could be helpful to deepen their understanding of screening procedures. Additionally, one participant described how the training reminded them to score screenings “*right away when it's fresh.”*

#### 3.1.2. Internalization

Internalization refers to motivations to implement a new practice, once participants have understood what the new practice entails (21). Motivations to engage in the training included an increase in patients with developmental support needs and reinforcement of previously learned concepts. Autism diagnosis and care management appeared to be important and timely skills to refine as one participant shared that they had “*been in practice for 25 years and I never had this many autistic kids”* and thus developmental care had “*really taken up a great part of my practice.”* Moreover, autism as a diagnosis had evolved considerably in the past few decades and this training allowed one clinician to “*completely [learn] some things that I had half learned… and I unlearned a couple of things that were… wrong… on top of experience with those evaluations 20 years ago… I learned how things had been refined.”*

#### 3.1.3. Individual specification

Participants' roles and backgrounds in developmental care varied but they seemed to fall into two groups, namely specialists who could use tools and strategies introduced in the training to strengthen current practices and non-specialists who were looking for ways to improve care for autistic patients as they wait for a specialist appointment.

One participant mentioned that they “*don't work with autism, so… [the training] was a way of getting… more than just book knowledge…”* They described a desire to be better able to recognize “*signs we might want to be noticing… And… how do I take that information from the trainings and put it into some practice?”* Several participants discussed patient education with one participant describing they were looking for *“tools and small things that we can support parents on doing to help their child in the interim [while waiting for Applied Behavior Analysis or ABA]?”*

#### 3.1.4. Communal specification

Participants discussed shared goals with colleagues, patients, and families. For example, one participant wanted to be better able to support parents who are “*expressing concern or follow[ing] through on the conversation the pediatrician is having. So, we are not diagnosing but we’re at least being able to provide bidirectional information.”* Participants described ways to improve their understanding of other staff roles. One participant expressed a desire to be “*able to think and understand how pediatricians approach a patient,… What could we do? What could we hold with them so they’re not holding it alone?”*

### 3.2. Cognitive participation

Participants engaged in problem identification, collaborations required to solve such problem, and discussed sustainability of the new practice. Participants identified the gap between the current and new practice, reorganized their work accordingly, and reflected on their ability to implement the new practice.

#### 3.2.1. Initiation

Participants reflected on the gap between their current practice and new strategies introduced in the training. When asked about potential care improvements, one participant “*foresee[s] that the majority of the time, when we have a child who has some developmental or other concerns that bring autism…, the child would have an answer or at least a next-step plan within two months.”* Another participant reflected on expanding their clinic's autism screening capacity by “*dedicat[ing] some sort of FTE [full time equivalent] resource.”* This was plausible because their clinic was larger “*compared to the other health centers…”* and thus could “*make decisions that smaller health centers can't make.”*

#### 3.2.2. Enrollment

Participants discussed how they collaborated and learned, such as having autism screening trainings and separate groups to target unique aspects of autism diagnosis and care. One participant shared that their clinic was “*doing a training… [for] our primary care providers in the RITA-T [Rapid Interactive Screening Test for Autism in Toddlers] screener.”* Similarly, another participant shared their clinic's desire to have PCCs “*… doing [developmental] tests”* in-house before making a specialist referral, which may involve long wait times.

#### 3.2.3. Legitimation

Participants discussed experiencing increased confidence in administering and advocating for autism screenings as they reflected on the value of strategies taught in the training. One participant “*did start doing CARS [Childhood Autism Rating Scale] evaluations after the [training] started, partially because I just had more confidence in doing it.”* Another participant described how PCCs in their workplace “*are increasingly confident in using the autism word…”*

#### 3.2.4. Activation

Participants described their decision in enacting strategies introduced in the training. Specifically, how these strategies fit into clinic workflow by allowing PCCs to collaborate more efficiently and getting crucial steps in autism care done promptly. One participant shared that their clinic “*ha[s] gotten more efficient, and the diagnosis is done quicker…”* and “*the referral… through the community health worker team is happening faster.”* The knowledge of different services needed for different age groups was also conducive to efficient care as described by a participant who was “*better able to talk about what [services are] available in the moment…”*

### 3.3. Collective action

Participants shared about implementing strategies introduced in the training within the context of current systems (e.g., referral process), barriers (e.g., the pandemic), time and resource allocations, and systems of accountability (e.g., staff roles and responsibilities).

#### 3.3.1. Interactional workability

Autism diagnosis and care involve multiple collaborators (e.g., school, insurance agency). The efficiency of interactions with such collaborators affected the implementation of strategies introduced in the training. Barriers included long wait times, complexity of electronic medical record (EMR) systems, billing delays, and limited bandwidth.

One participant described “*bewildering random delay as to whether… billing agencies will accept [autism] diagnosis.”* Even when patients “*have the same [government] insurance”* and presented with “*the same [clinician] doing the same test with the same letter template,”* they may receive varying responses based on the agencies' understanding of whether they would receive reimbursement. Additionally, EMR systems were described as “*not talking to one another… So, if you refer to developmental peds [with a different EMR system], I’m not sure how quickly you’re actually being able to see [the referral].”* Consequently, timely referrals did not always result in timely services.

#### 3.3.2. Relational integration

Participants discussed structure and accountability within clinics needed for successful referrals to long-term services such as behavior therapy. One participant “*would love someone to walk [families] through this [ABA] process because… you see [families] time and time after that and you’re like ‘Where are you in the process?’ ‘Oh,… we haven't really started or… we got stuck at paperwork which is… the very beginning [step].’”* This issue did not only occur with ABA, as the participant continued that they “*refer[red] a patient to EI [Early Intervention] and then like nothing happens.”*

#### 3.3.3. Skillset workability

Participants described the distribution of responsibilities in implementing a new practice. Expanding training for autism screening beyond specialists could be key to timely referrals and care. One participant said, “*If [screening and referral] was all in the hands of one person to…, it can become unwieldy and time intensive,”* causing the “*diagnostic journey [to take] eight months… of [the child's] development.”* Multiple participants mentioned that it was helpful to have other PCCs who were able to conduct screenings. One participant felt “*very fortunate to have [two colleagues conduct screenings]”* as it “*helped… take some of the stress of the long wait of getting an evaluation in our developmental clinic.”*

#### 3.3.4. Contextual integration

Participants discussed barriers to autism screening and care such as gaps between appointments in pediatric patients. One participant described that “*[pediatric visits] schedule goes from every two months to… every year… very quickly….”* Due to age restrictions for some services, “*that kid [becomes] too old for EI, [but] too… young for school.”* Combined with “*specialists already hav[ing] a one year waiting list,”* the participant expressed a desire to “*build something in our practice to catch [kids waiting for services].”*

Another barrier discussed was “*time, right?… It's obviously really hard to… get a full picture of a kid's development in… 15 min[s].”* It is particularly challenging when “*a full autism evaluation”* takes *“an hour.”* Lastly, limited staffing and resources was cited as a barrier by a participant whose clinic consisted of “*only three pediatricians and me… and we are teeny tiny.”*

### 3.4. Reflexive monitoring

Individuals described how they evaluated and perceived the utility of strategies introduced in the training. Additionally, participants shared their evaluation of the training delivery and logistics.

#### 3.4.1. Systemization

Participants described how strategies introduced in the training impacted efficiencies of their practice. These changes were reported through informal observations. After the training, one participant “*just realizing… importance [of documentation] and trying to make maybe some flow changes or systematic changes to help us to really not miss these key… screeners.”*

#### 3.4.2. Communal appraisal

Participants shared how they evaluated a new practice as a group. Participants discussed how the training provided an opportunity to come to a shared understanding of best practices with other clinicians. One participant shared that “*it's always nice to hear about how things run differently in different clinics….”* Learning more about other clinics also created a “*community feel I thought was great.”*

#### 3.4.3. Individual appraisal

Participants reflected on how the training benefitted their individual practice. One participant “*valued… the case presentations”* because it was “*that one level up”* from didactic lectures. Another participant “*loved the videos… I thought they were just such a great teaching tool”* in a virtual environment. Participants also shared recommendations to improve future trainings, such as having more training in addressing “*that lag time and the desperation of parents”* and “*… little interventions or pearls that we can share with parents…”*

#### 3.4.4. Reconfiguration

Participants reflected on current or planned changes to their care for autistic patients after the training. One participant recalled “*ma[king] a point of using the word [autism]….”* Another participant shared that the training had “*brought to our primary care practice like a renewed focus on autism.”* The enhanced understanding of best practices “*made us even less tolerant of the wait times and… doing more work to… move heaven and earth to not let the wait times be a barrier for our patients.”*

## 4. Discussion

In this study, NPT was instrumental to organize qualitative data into actionable recommendations for a virtual PCC training program on autism screening and care. Qualitative data analysis revealed participants' motivations, attitudes, and perceived barriers regarding autism screening and care management strategies taught in the training. NPT was useful in highlighting both the process (training delivery) and outcome (practice change) aspects of evaluation.

Participants discussed the **coherence** construct or understanding of strategies introduced in the training. The training gave participants ideas to improve care by scoring autism screenings sooner, adapting behavioral observations based on patients' age, and providing more in-house care while waiting for external services. Benefits of early autism diagnosis and support are well established (27, 28) yet wait times for developmental-behavioral services in recent years grew due to increased demand (29) and disruptions from the COVID-19 pandemic. Enhancement of in-house care could help bridge this gap (29). Practice recommendations include expansion of topics relevant to primary care (e.g., feeding, sleeping, medication dosages) and having a centralized location for resources about local services that PCCs can share with each other and patients/families.

Participants were also motivated by an observed increase in patients needing developmental care. Child mental health care was declared as being in a state of crisis partly due to the decline of services available and workforce capacity (2). Additionally, senior clinicians reported wanting to refine their knowledge about autism given recent major changes to autism as a diagnosis (30). Although comprehensive and systemic changes are needed, equipping clinicians with up-to-date knowledge could be a step in bridging gaps in services (31). Strategies that could help expand screening capacity included hands-on opportunities for clinicians to practice autism screening and modelling use of autism screening tools on real patients.

Participants also discussed components needed to implement strategies introduced in the training, which were coded using the **cognitive participation** construct. First, increasing the number of PCCs able to administer and advocate for autism screening could increase access, which aligns with past research (31). Participants described increased confidence in administering autism screening and educating families about autism, which in turn led to increased efficiency. Expansion of autism screening and care could address barriers to timely autism evaluation especially in low resource populations such as those served by this study's participants (32).

Second, a shared understanding of goals and accountability was needed as autism service referrals often involved multiple collaborators (e.g., behavior therapy providers, insurance agencies). Therefore, participants also expressed a desire for stronger understanding of how different services work, particularly EI and ABA as the latter is regarded as the golden standard for autism treatment (33). Including external experts on autism-related care could enhance clinicians' knowledge and establish connection with key collaborators. Past studies indicated that improvements were needed in follow up care of children who screened positive for autism (34, 35). Although additional research is needed to examine factors affecting low referral rates to follow up services (35), increased understanding of services may enhance PCCs' ability to navigate and advocate for timely service receipt.

Additionally, participants reflected on action steps they took to implement strategies introduced in the training, which were coded using the **collective action** construct. Barriers to autism screening and care were also discussed such as gaps between pediatric visits, EMR complexities, and inconsistent insurance requirements, all of which could contribute to service delays and aligned with prior research (9). Some barriers were outside clinicians' control, yet participants were committed to improving care whenever possible. Considerable investment is needed to expand the developmental-behavioral pediatric workforce and services (36), but enhancing aspects of care within a clinician's control may be one step closer to short-term care improvement.

For example, easy access of screening materials (e.g., printouts in exam rooms, centralized location of digital copies), clear follow-up protocols with defined responsibilities such that screenings have actionable outcomes, and enhanced patient education. Enhanced longitudinal family-clinician rapport that could result from these care improvements may further facilitate family's engagement with clinician recommendations, as found within the context of Latinx families (37).

Lastly, the training was well-received as evident from transcript text coded using the **reflexive working** construct. Participants cited the feeling of community with other attendees. Connection with other clinicians were found to be facilitators of clinician well-being (38, 39), which is crucial to maintain for an increasingly strained workforce (40, 41). The live, synchronous format of the training where trainers could engage attendees in real time appeared to be key in fostering such connection. Moreover, participants reported renewed focus on autism and decreased tolerance of wait times as they now had the tools and strategies to help remedy the situation in the short-term.

There were several limitations to this study. First, there was limited transferability of findings due to small sample size (*n* = 10) and self-selected participants from an urban area in northeastern United States. Participants, however, represented most of the eligible health centers (85.7%) with varying patient populations. Moreover, there was a large range in years of experience despite similar levels of prior interest in autism. Second, clinicians who participated in focus groups may be subject to social desirability bias as many knew and worked with each other. Findings however were gathered from a mix of focus groups and personal interview data.

### 4.1. Utility of NPT

NPT provided a helpful framework to organize qualitative data into actionable recommendations. Qualitative data analysis revealed participants' motivations, attitudes, and perceived barriers regarding autism screening and care management strategies taught in the training. Findings could guide efforts to enhance and sustain implementation of autism screening and care management in diverse urban settings. NPT could also be used to guide evaluations of other clinician training programs in addition to implementation of specific protocols in healthcare settings (23).

Similar to findings of a review of studies using NPT, we found overlaps between NPT constructs that made it difficult to assign a single construct to our data (22). For example, activation under cognitive participation and individual appraisal under reflexive working. Activation occurred when participants decided to enact a new practice whereas individual appraisal occurred when participants evaluated the value of a new practice after they had enacted it (21). However, participants tended to discuss these topics simultaneously; their decision to enact a new practice was implied by their reasoning involving the value of such practice. Although the overlap did not impact actionable recommendations out of the analyses, it is important to consider when using NPT.

Moreover, because participants varied in their baseline knowledge and experience with autism care, the novelty of their practice change and whether it was influenced by the training was unclear. As such, it was difficult to assign data to the construct differentiation under coherence, which referred to the contrast between existing vs. new practice. Another construct that was challenging to apply was systemization under reflexive working, which referred to participants' way of evaluating a new practice (21). The definition could apply to both formal (i.e., quality improvement studies) and informal (i.e., conversations) evaluation methods, but participants tended to share their thoughts about the training without sharing specifically how they gathered data to come to their conclusions. It was largely implied that they evaluated the training through personal observations and informal conversations with colleagues. Lastly, the current study focused on two innovations, namely the tools and strategies introduced in the training as well as the training delivery itself (i.e., virtual, year-long, inter-professional training). Careful attention was paid to specify which innovation codes applied.

## 5. Conclusion

PCC training on autism screening and care management could potentially address service access issues. There were distal barriers outside of a clinician's control, but equipping clinicians with knowledge and self-efficacy about autism care may help address proximal barriers within their control. NPT allowed for detailed assessment of process and outcome evaluations for a PCC training program, identification of gaps, and practice recommendations. Moreover, NPT was useful in highlighting both the process (training delivery) and outcome (practice change) aspects of evaluation and providing a framework for delivering recommendations to program implementers. Lastly, NPT could be used as a guiding framework for other clinician training programs, however, defining the new practice of interest may need to be further clarified when working with a participant group with varying baseline knowledge.

## Data Availability

The authors cannot provide raw data per IRB regulations. Further inquiries can be directed to the corresponding author/s.

## Appendix A. Focus group guide with sample questions/probes

### Part I: individual-level application of ECHO Autism strategies (Suggested time: 15 minutes)

1. What skills are you applying, meaning what are you able to do differently because of what you learned from the ECHO Autism Program? Are any skills missing from what you received in the ECHO Autism Program? *(Probe for screening, referral, treatment)*
2. What knowledge did you gain, meaning what did you learn that you can apply to your practice and relay information to your patients? Are any pieces of knowledge missing? *(Screening tools, referral protocols, resources*)
3. How did the program change your perspective on the education and care of your patients? *(Probe for comfort of using terminology, confidence in referring, managing care)*

### Part II: clinic-level application of ECHO Autism strategies (Suggested time: 15 minutes)

1. How has your clinic’s practice changed because of your participation in in the ECHO Autism Program? Who have been implementing these changes? *(Probe for administrative support, connection/community within the practice)*
2. What are some of the benefits to implementing the strategies from the ECHO Autism Program training given your current clinic context? Who are the recipients of these benefits? (*Probe for EMR supports, protocols for referral and screening, sense of community*)
3. How do you determine if strategies from the ECHO Autism Program training are working for your clinic? *(Probe for patient satisfaction, staff satisfaction, burnout factors (e.g., feeling worthwhile at work, work is satisfying/meaningful, they are contributing professionally in ways they value)*

### Part III: opinions about the ECHO Autism training (Suggested time: 10 minutes)

1. What parts of the ECHO Autism Program training do you value the most? (*Probe for parts that can be transferrable to other clinics and avenues.*)
2. What could have been done differently during the training to better meet the learning objectives? Reflect on your experiences with case presentation and discussion. (*Probe for support from clinic administration, further resources, etc.*)
